# Coarse-Grained Molecular Dynamics Simulations Reveal Potential Role of Cardiolipin in Lateral Organization of Proteorhodopsin

**DOI:** 10.1021/acs.biochem.4c00831

**Published:** 2025-03-26

**Authors:** Alexander Wroe, Eric Sefah, Blake Mertz

**Affiliations:** C. Eugene Bennett Department of Chemistry, West Virginia University, 100 Prospect Street, Morgantown, West Virginia 26506, United States

## Abstract

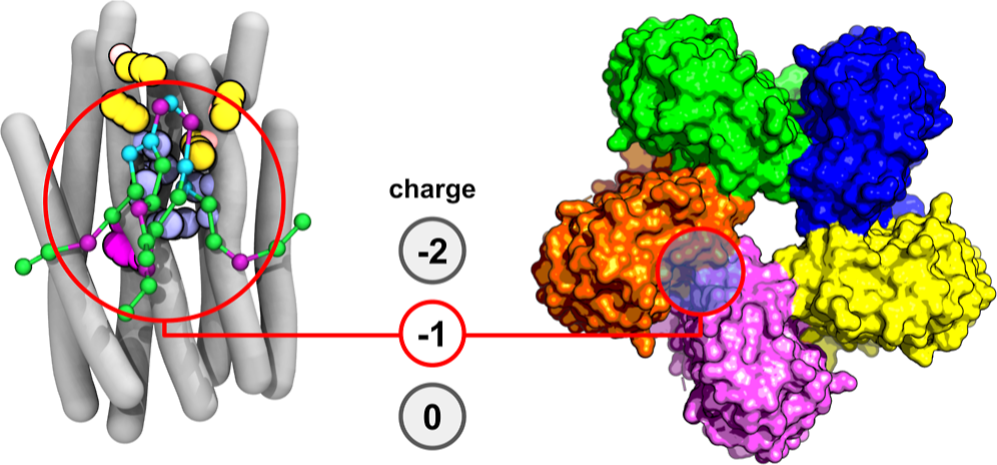

Proteorhodopsin (PR) is a microbial light-harvesting proton pump protein that is ubiquitous in marine ecosystems and is critical for biological solar energy conversion. A unique characteristic of PR is that its function can be directly affected by changes in the surrounding cellular membrane environment. Cardiolipin (CL) is a commonly found lipid in mitochondria and bacterial cell membranes and plays a prominent role in the function of numerous integral membrane proteins, due to its bulky conical shape and ionizable nature of its headgroup. CL can directly interact with other microbial rhodopsins and modulate their function; however, the potential role of CL in the function of PR is unclear. In this study, we used the MARTINI coarse-grained force field to characterize the interactions of CL with PR in a model bilayer via coarse-grained molecular dynamics (MD) simulations. Our simulations show that both electrostatic and nonpolar forces drive residue-specific interactions of CL with proteorhodopsin, especially for the asymmetrical −1 charge state of CL. Several CL binding sites were identified, with lipid–protein interactions occurring on the μs time scale. These binding sites are proximal to key functional areas and regions of oligomerization on PR, suggesting that CL could play a role in modulating proton pumping of proteorhodopsin.

## Introduction

**Figure 1:**
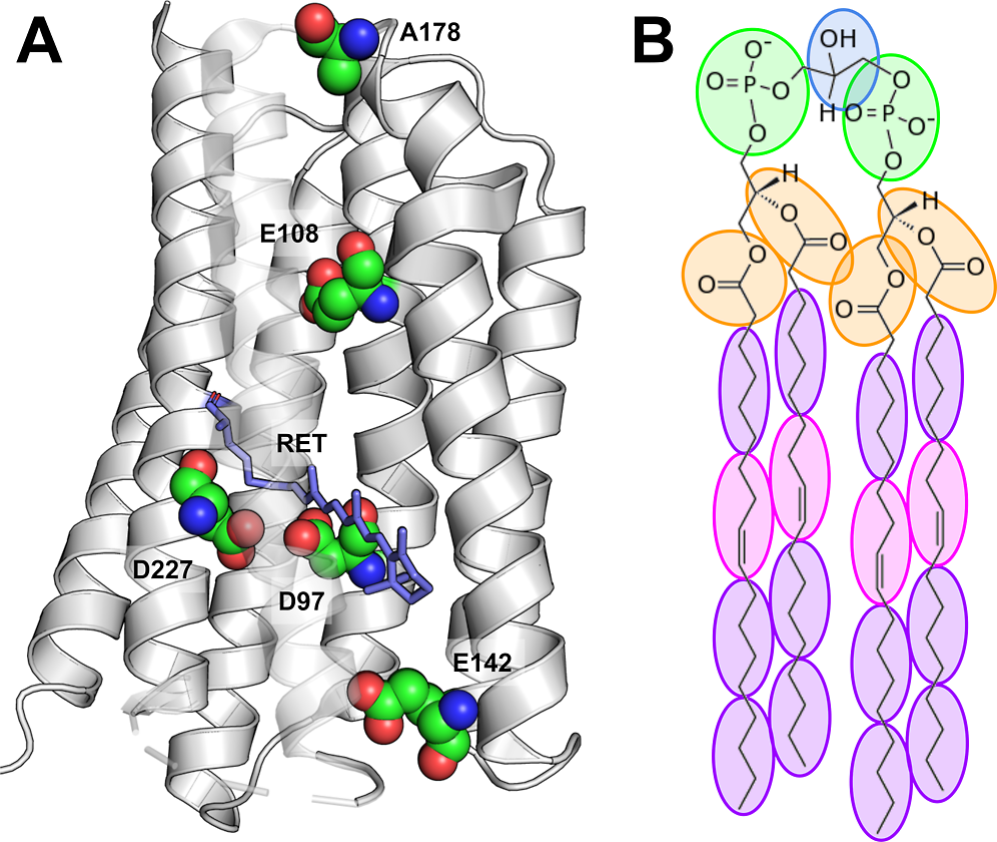
Molecules used in this study. (A) Proteorhodopsin, a microbial proton pump. Structure of green proteorhodopsin (PDB 7B03). Important residues to the function of PR are highlighted. Ribbons: PR secondary structure; spheres: residues including side chains; sticks: retinal chromophore. (B) Chemical structure of 18:1 cardiolipin. Coarse-grained topology is shown by colored circles.

Proteorhodopsin (PR) is one of the most ubiquitous microbial proteins, having spread via horizontal gene transfer to bacteria, archaea, and eukaryotes.^1−4^ PR is a heptahelical membrane protein that functions as a light-driven proton pump; photoactivation occurs when the retinal chromophore absorbs a photon, leading to an all-trans → 13-*cis* isomerization.^5^ Light-harvesting efficiency has prompted the protein to adapt to environmental conditions (e.g., depth in the ocean), leading to blue and green-light absorbing PRs.^6^ Residues critical to proton pumping in PR are conserved among other microbial rhodopsins, including the retinal Schiff base linkage (K231), the Schiff base counterion complex (D97 and D227), proton donor (E108), and putative proton release group (E142).^7^ In addition, the spectral wavelength of absorption can be shifted with substitutions at position 105 (L105Q) and 178 (A178R)^8,9^ (Figure 1A).

A particular characteristic of PR that has drawn increasing interest is the relationship between membrane environment and protein function: solvating PR in detergent micelles versus lamellar lipid environments can tune the oligomeric state of PR from monomeric to pentameric and hexameric oligomers, which in turn affects proton pumping efficiency via a shift in p*K*_a_ of the PR photocycle.^10,11^ The oligomer interface is primarily along helices A and B,^12−14^ with the EF loop playing a critical role in proton uptake.^15^

Cardiolipin (CL, 1,3-bis(*sn*-3′-phosphatidyl)-*sn*-glycerol) is a commonly found lipid in mitochondrial and microbial membranes, comprising at least 20 and 5 mol %, respectively.^16−18^ CL has a symmetric topology consisting of two phosphatidic acids conjugated by a glycerol backbone^19^ (Figure 1B). The twin phosphate headgroup allows CL to adopt three charge states (neutral, −1, and −2), with a preference for the −2 charge state at neutral pH.^20^ This structure gives CL unique physicochemical properties: its multiple protonation states are hypothesized to allow CL to act as a buffering agent for changes in pH proximal to proton pumping membrane proteins (e.g., ATP synthase, translocon, PR)^21,22^ and its conical shape imbues membranes with intrinsically negative curvature and enhanced fluidity.^23,24^ CL also functions as a ligand for membrane proteins; it acts as a molecular glue for the formation of supercomplexes in the mitochondrial electron transport chain and bacterial translocon^25,26^ as well as increasing ATP turnover. In particular, CL has three primary means of interacting with proteins to form stable protein–lipid complexes: (1) the phosphate head groups can form salt bridges between positively charged side chains; (2) the central hydroxyl group can hydrogen bond with neutral phosphate or glycerol groups; and (3) the acyl tails associate via hydrophobic forces with nonpolar side chains.^27^

Several cases in microbial biology point toward a potential relationship between PR and CL. X-ray crystallography identified specific binding sites of CL to the bacterial photosynthetic reaction center (PRC),^28^ and spectrophotometric studies showed that the presence of CL enhances electron transfer,^29^ while other studies on CL in mitochondria show that the lipid interacts with multiple proteins in the electron transport chain.^30−34^ In addition, glycocardiolipion, an archaeal analog of CL, binds specifically to the canonical microbial proton pump, bacteriorhodopsin.^35^

Molecular dynamics (MD) simulations, in particular coarse-grained MD simulations,^36,37^ have emerged as an invaluable tool in biophysically characterizing specific interactions between lipids and membrane proteins.^38^ In this study, we used coarse-grained MD simulations to model a proteolipid system consisting of green PR and CL in all possible charge states. We identified two putative binding sites where CL interacted with PR on the microsecond time scale; these binding sites overlay with functional hotspots on PR and may provide clues to the role that CL plays in optimizing the proton pumping function of this microbial rhodopsin.

## Methods

### Simulation Setup

Proteolipid systems of monomeric green PR were created for each 18:1 CL charge state (CL-0, CL-1, and CL-2) at three different mole fractions (0%, 5%, and 10%), with the remaining membrane lipids comprised of 1-palmitoyl-2-oleoyl-*sn*-glycero-3-phosphoethanolamine (POPE) and 1-palmitoyl-2-oleoyl-*sn*-glycero-3-phosphoglycerol (POPG) in a 3:1 POPE:POPG mole ratio. Bulk solvent for each system was approximately 3500 polarized water molecules, 20 chlorine ions, and 50 sodium ions. Lipid-only systems were constructed in the same manner. Systems were constructed with the Insane tool for MARTINI.^39^ The force field parameters for PR, POPE, POPG, ions and water were taken from the polarizable force field MARTINI v2 to assess the effects of the cardiolipin charge states most accurately,,^36,40,41^ of which the most recent MARTINI parameters were from Dahlberg et al.^42^ MARTINI v2 was chosen over v3 due to the fact that the CL parameters were developed to be compatible with v2, and the polarizable water model was chosen over the single-point model to more accurately model the electrostatic interactions with the charged environment of the CL molecules. Initial coordinates and topology for green PR were generated from the solution NMR structure.^43^ All simulations were performed in GROMACS 5.1.2. Minimization and equilibration started with a 2 fs time step with force constraints applied to the entire system. These steps consisted of 2 minimization cycles and 5 equilibration runs with a length of 20,000 steps, each with an increase in time step (5, 10, 15, 20), while the force constraints on the entire system started at 1000 and were halved each step and completely released at the 20 fs time step, additional force constraints of 200 were applied to lipid head groups in the initial equilibration run and reduced in the same manner so that no force constraints were present during the production runs. All equilibration simulations used a semi-isotropic Berendsen barostat^44^ at 1 bar and a velocity rescaling thermostat at 303 K. Production simulations used the Parrinello–Rahman barostat^45^ at 1 bar and the velocity rescaling thermostat at 303 K with a 20 fs time step. All production runs were for 10 μs.^46,47^

### Analysis

The first 2 μs of each trajectory were removed to account for the decorrelation time of the system. Binding site analysis was carried out using the PyLipid package.^38^ A dual cutoff of 5 and 7 Å was used to quantify CG bead contact, and residence times were calculated as 1/*k*_off_ with a survival time correlation function σ(*t*)
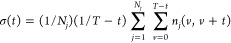
1with *T* as the total length of the simulation, *t* as the current time, *N*_*j*_ is the total number of contacts, and the last term is a binary function that is equal to 1 when the contact of lipid *j* lasts between time *v* and *v* + 1 and is equal to zero otherwise. The survival time correlation function was then plotted as a biexponential model to represent long and short lipid relaxation times
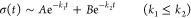
2where *k*_1_ is assumed to be the lipid dissociation constant, *k*_off_. Binding sites were identified using Louvain’s method of calculating network community structures, which partitioned the network of residues into discrete communities representing individual binding sites. Statistics for all binding sites for all combinations of CL charge states and mole fractions are provided in the Supporting Information (Tables S1–S6).

## Results

### Characterization of Membrane Behavior

**Figure 2:**
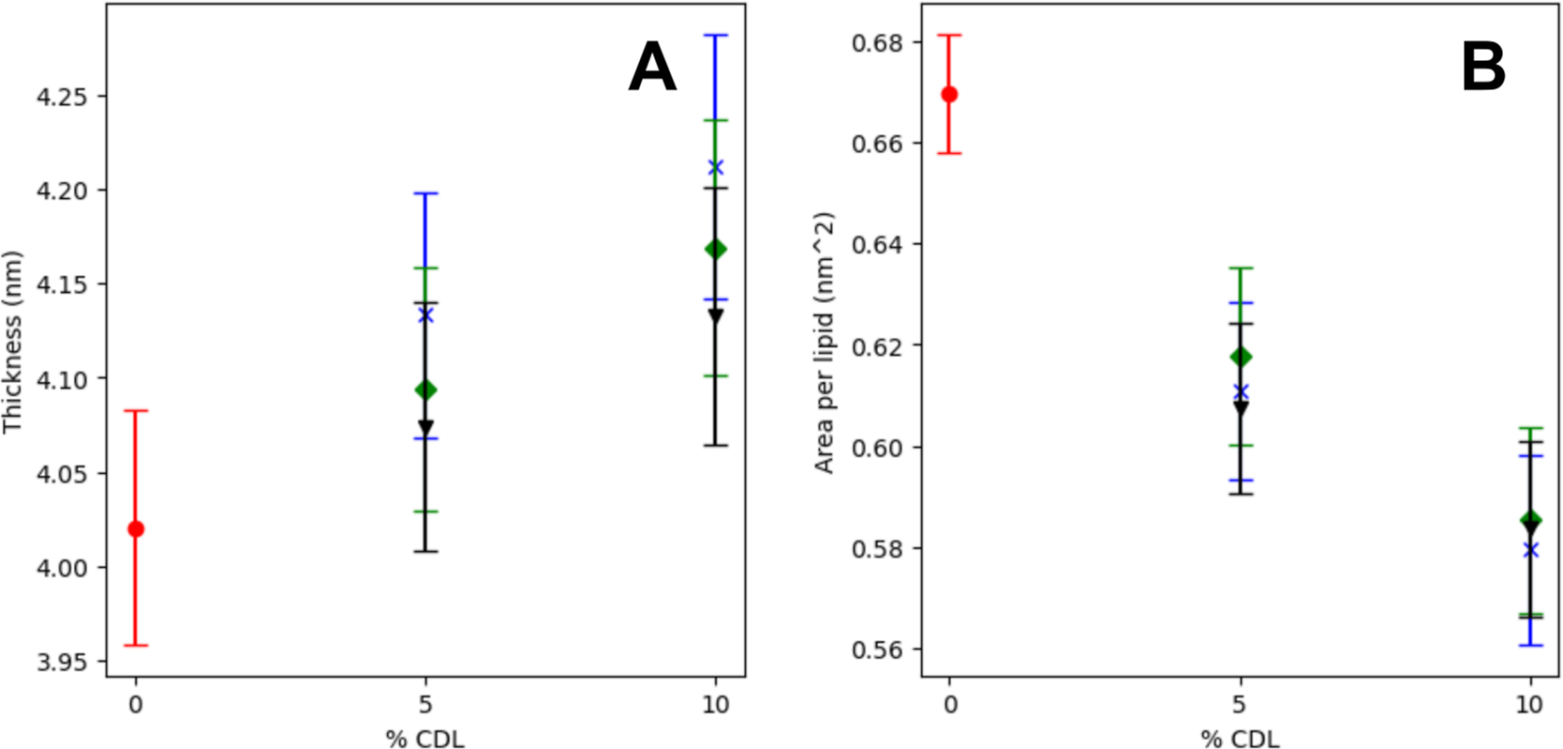
Cardiolipin increases lateral packing of the lipid bilayer. (A) Average membrane thickness and standard deviation as a function of mol % of CL. (B) Average area per lipid and standard deviation as a function of mol % of CL. Red circle: no CL; blue cross: CL-0; green diamond: CL-1; black triangle: CL-2.

Analysis of membrane thickness and area per lipid (APL) showed that CL concentration impacted both metrics: increases in CL concentration is inversely correlated with APL and is directly correlated with membrane thickness (Figures 2 and S1). However, this correlation is not perfectly linear as a function of the charge state of CL: at 10 mol % CL, both CL-1 and CL-2 have equivalent APL, yet CL-2 has less of an effect on membrane thickness. Order parameters for each charge type and mol % of CL show that the −2 charge state leads to more ordering of CL compared to the −1 and neutral state (Figure S2). This ordering effect propagates to the rest of the bilayer in the form of APL and thickness, yet does not affect the conformational organization of individual POPE and POPG lipids as shown by their respective order parameters.

### Proteorhodopsin Favors Interactions with C

**Figure 3:**
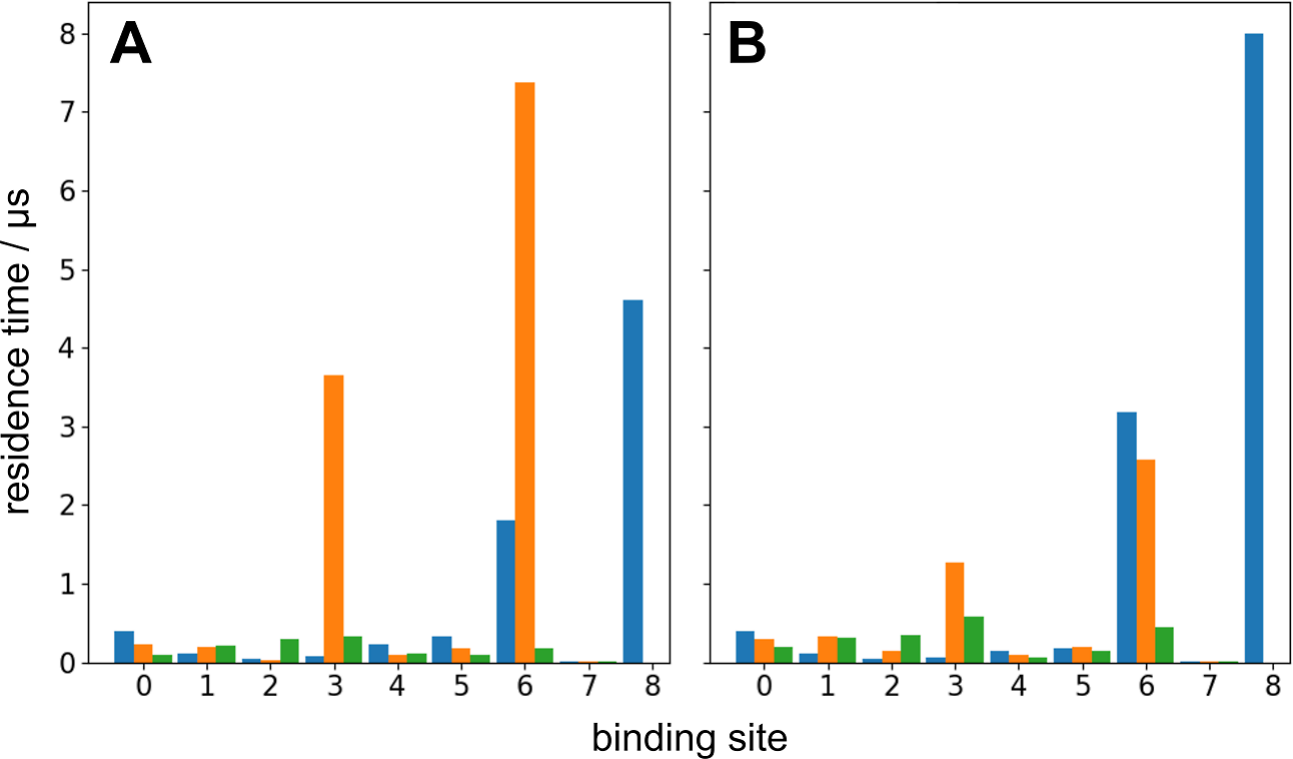
Charge state of CL has a direct impact on effectiveness of binding to PR. (A) CL residence times for individual binding sites with 5 mol % CL as a function of CL charge (0, −1, −2). (B) CL residence times for individual binding sites with 10 mol % CL as a function of CL charge (0, −1, −2). Blue: CL-0; orange: CL-1; green: CL-2.

LEach bilayer lipid type was able to stably interact with the membrane-exposed surface of PR, albeit at significantly different time scales. For CL, nine distinct binding sites were identified, with all but one being shared between the three charge states. For the other lipids, POPE had 11 possible binding sites while POPG had ten. A comparison of the binding site residence times shows that in general, all three types of lipids interacted with PR on the subμs time scale (typically less than 100–200 ns) (Figure 3 and Tables S7 and S8). However, for CL, three binding sites—BS3, BS6, and BS8—had residence times >3 μs.

**Figure 4:**
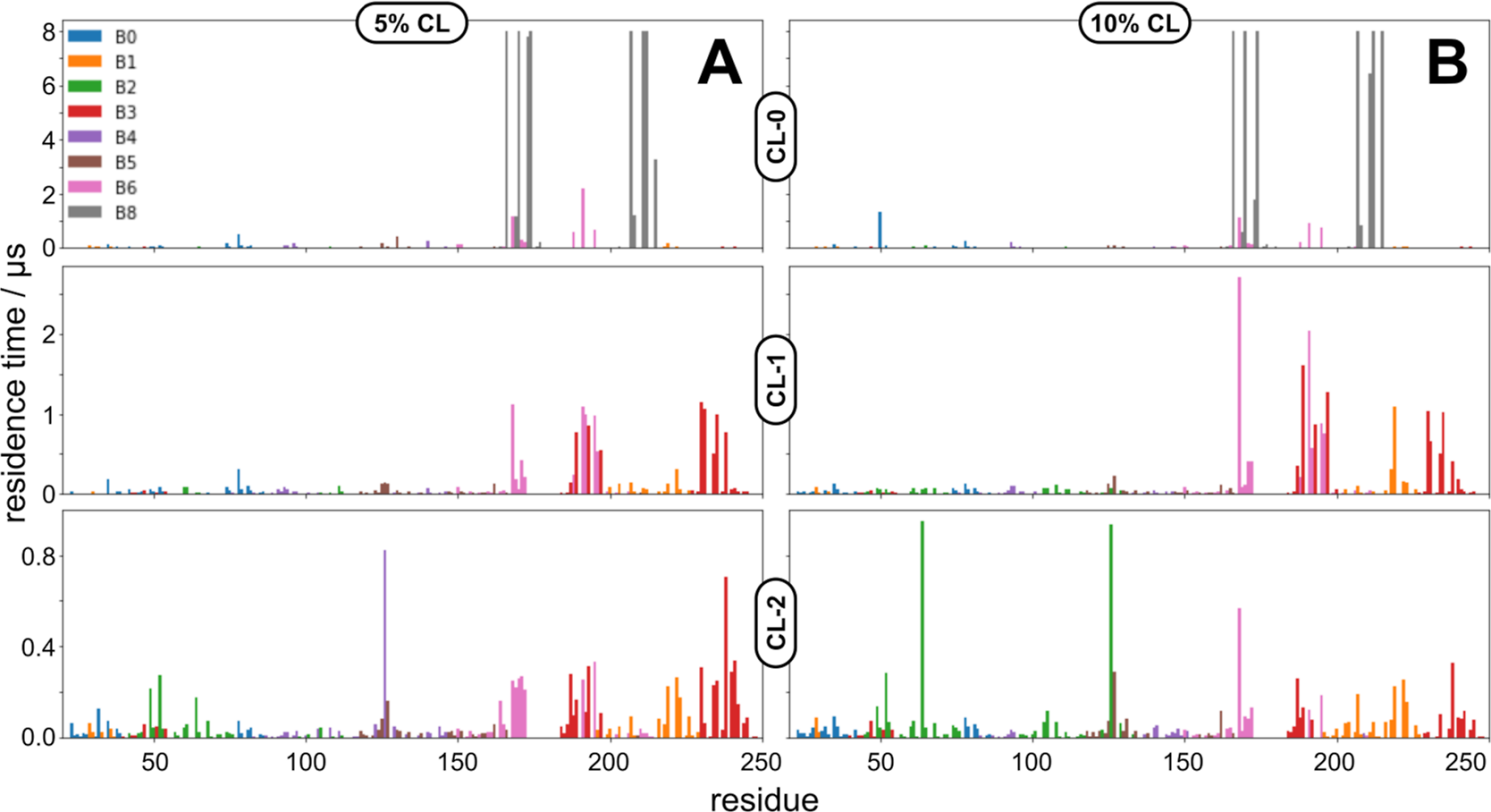
Per-residue residence times show a mix of distinct interactions and overlap of residues that are involved in binding of CL to PR. (A) Per-residue residence time of CL at 5 mol % CL and a function of charge state of CL. (B) Per-residue residence time of CL at 10 mol % CL and a function of charge state of CL.

Once we start to account for changes in the concentration of CL, different trends emerge, particularly for binding sites with μs residence times (BS3, BS6, and BS8) and with specific charge states of CL. Long residence times for BS3 are specific to CL-1 but markedly decreases when increasing from 5% to 10% mole fraction of CL (Figures 3 and 4). One possible explanation for this is that the asymmetric charge topology of CL-1 allows for nonbonded interactions (e.g., a hydrogen bond or salt bridge) to form between CL-1 and a particular residue in PR (more discussion on this below). However, it is likely that this nonbonded interaction is not strong enough to overcome crowding from additional CL molecules, which is why we observe a decrease in residence time at BS3 from 3.8 to 1.3 μs when going from 5 to 10 mol % CL. In contrast, BS6 may utilize both polar and nonpolar interactions to stabilize CL binding to PR. The residence time for CL-0 increases as a function of the mol % of CL, indicating that shape complementarity is key to lipid binding. The more CL that is available, the more lateral interactions can occur between CL and BS6 of PR. On the other hand, the residence time of CL-1 at BS6 sharply decreases as a function of the mol % of CL—the asymmetric charge surface of the CL-1 headgroup has specific charged interactions with BS6, but these interactions are not strong enough to overcome increased CL–CL headgroup interactions at the 10% mole fraction. Finally, BS8 is completely specific to CL-0, and increases with increasing amounts of CL. However, the fact that a low-propensity charge state of CL combined with the location of BS8 is in the middle of the hydrophobic region of the membrane make it unlikely that this binding site is a true binding site for CL.

### Detailed Characterization of Stable Binding Sites

**Figure 5:**
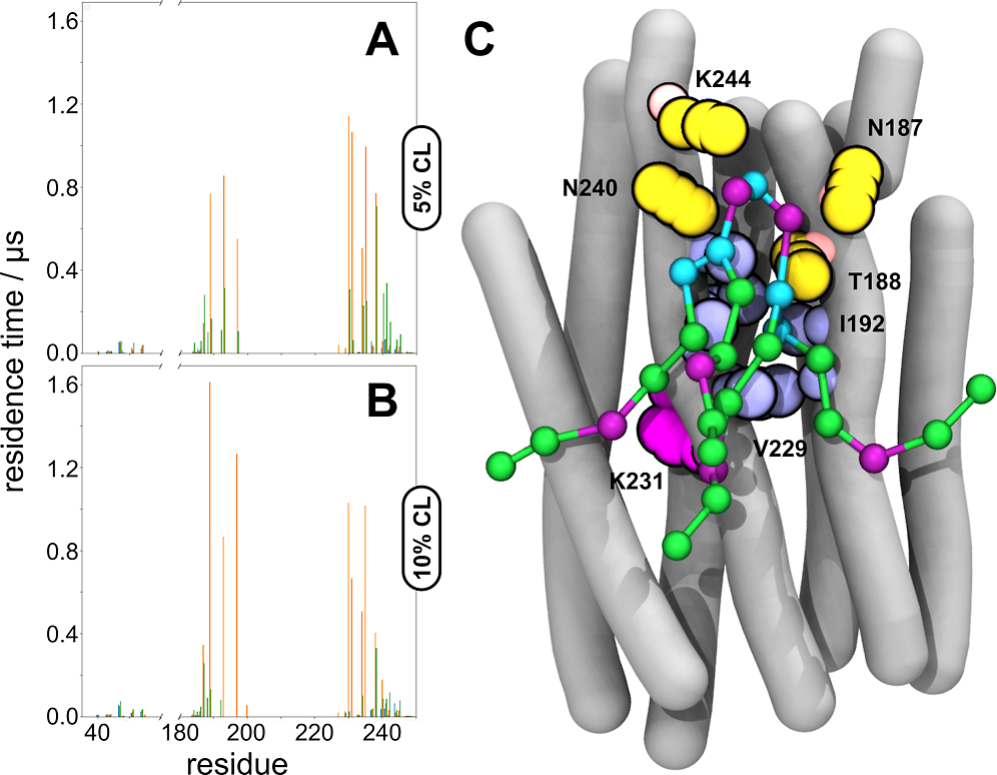
Asymmetric headgroup topology of CL in −1 charge state drives long-time scale interactions with binding site 3 on PR. (A) Per-residue residence time of CL at 5% mole fraction as a function of charge state (0, −1, −2). (B) Per-residue residence time of CL at 10% mole fraction as a function of charge state (0, −1, −2). Green: CL-0; orange: CL-1; blue: CL-2. (C) Representative snapshot of CL binding to PR at binding site 3. Yellow spheres: polar residues in BS3; purple spheres: nonpolar residues in BS3; magenta spheres: D227 and K231; balls and sticks: CL-2; tubes: PR secondary structure.

Binding site 3 (corresponding to CL-1 at 10 mol %) is the largest binding site at approximately 23 nm^2^ (Figure S3) and is located on the cytoplasmic side of helices F and G. The side chains of residues within BS3 lie on the outside face of the retinal binding pocket (i.e., D227 and K231), with a dual topology that facilitates strong binding to both the headgroup and acyl chains of CL-1 (Figure 5). Headgroup interactions are stabilized by N187 and T188 (helix F) and N240 and K244 (helix G), whereas the acyl tails have extended interactions with nonpolar residues including A226, F228, V229, I232, L233, and F234 (Table 1). Another noticeable behavior of CL-1 is its ability to interact laterally with a significant fraction of the hydrophobic surface area of PR despite being localized to BS3. The flexibility of the four acyl chains on the CL-1 molecule often leads to a splayed conformation in which they straddle the gap between helices F and A. This behavior could potentially interfere with the monomer–monomer interface that is localized to the salt bridge trimer (D50/R51/E52) that was first identified by Glaubitz and co-workers.^12^

**Table 1:** Average Per-Residue Residence Time for BS3 for CL-1

BS3	10% CL −1		5% CL −1	
residue	time/μs	*R*^2^	time/μs	*R*^2^
F46	0.050	0.967	0.050	0.999
Y186	0.145	0.999	0.345	0.999
T188	0.768	0.968	1.610	0.923
I192	0.856	0.977	0.867	0.984
G196	0.551	0.986	1.266	0.983
V229	1.143	0.937	1.030	0.917
N230	1.065	0.995	0.665	0.975
L233	0.507	0.997	0.507	0.931
F234	0.995	0.996	1.016	0.997
I237	0.770	0.995	0.405	0.986
W239	0.058	0.999	0.178	0.994
N240	0.071	0.997	0.064	1

**Figure 6:**
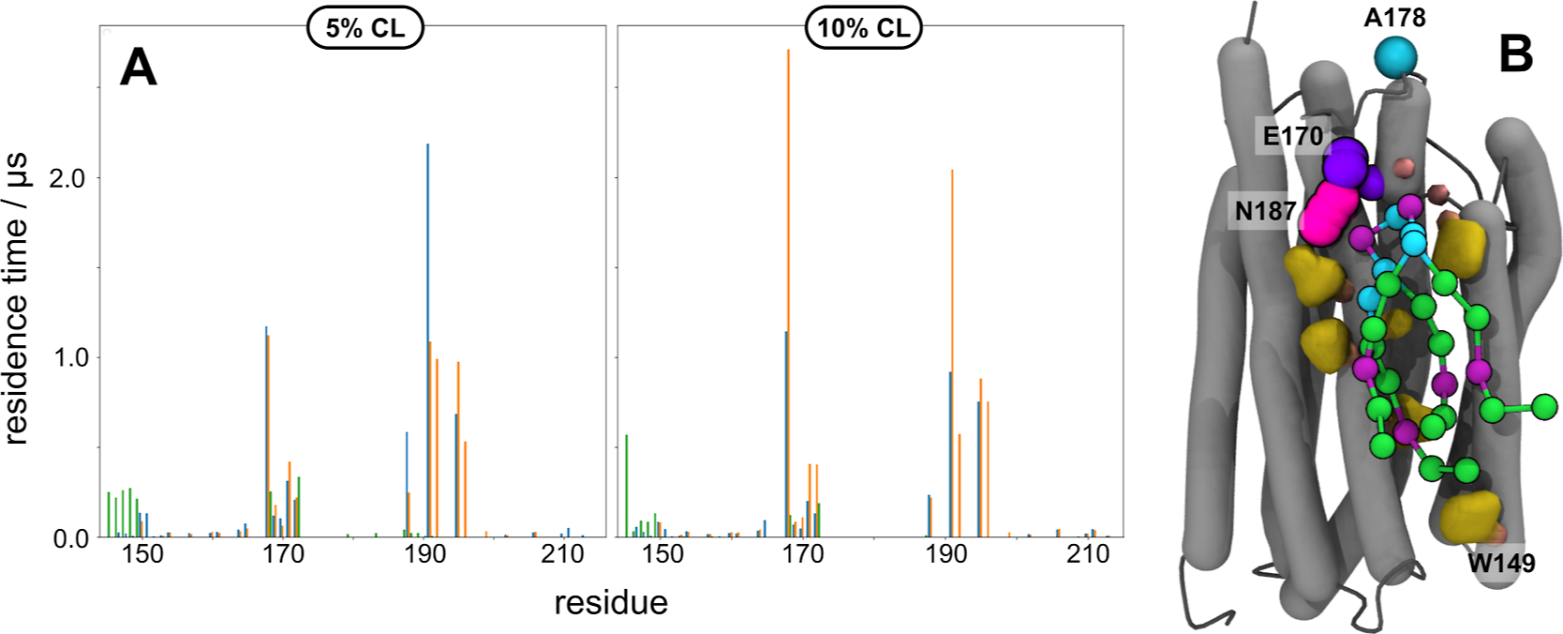
Binding site 6 is predominantly skewed toward binding of CL in the −1 charge state. (A) Left: per-residue residence time of CL at 5% mole fraction as a function of charge state (0, −1, −2) for binding site 6. Right: per-residue residence time of CL at 10% mole fraction as a function of charge state (0, −1, −2) for binding site 6. (B) Representative snapshot of CL binding to PR at binding site 6. Spheres: residues involved in color tuning of PR (A178) and polar nonbonded interactions (E170 and N187); yellow surface: nonpolar residues involved in binding of CL acyl chains in BS6; balls and sticks: CL.

Binding site 6 (also corresponding to CL-1 at 10 mol %) has a surface area of ∼17 nm^2^ and is proximal to the EF loop in PR, a region that has been implicated with control of the PR photocycle as well as tuning the spectral wavelength of absorption.^9,48^ Of the residues that make up BS6 (W167, A168, E170, and G171 on helix E and N187, M190, Y191, I194, and F195 in helix F), E170 and N187 facilitate a strong nonbonded electrostatic interaction. The two polar residues form a dyad that attracts the negatively charged headgroup on CL-1, with average residence times >0.4 μs at both 5 and 10 mol % (Figure 6 and Table 2). Multiple aromatic residues augment BS6 by providing a nearly uniform nonpolar surface to stabilize interactions with the acyl chains of CL-1, as evidenced by residence times up to 2.71 μs. In general, the asymmetric charge distribution of the CL-1 state appears to favor binding of CL to both BS3 and BS6; the orientation of the CL headgroup is distinctly shifted in the −1 charge state (Figure S4).

**Table 2:** Average Per-Residue Residence Time for BS6 for CL-1

BS6	10% CL −1		5% CL −1	
residue	time (μs)	*R*^2^	time (μs)	*R*^2^
W149	0.088	0.999	0.081	1
W167	1.121	0.999	2.71	0.949
A168	0.178	1	0.084	1
G169	0.061	1	0.108	1
E170	0.419	0.999	0.404	0.999
G171	0.217	1	0.402	1
N187	0.245	1	0.218	0.986
M190	1.087	0.997	2.042	0.984
Y191	0.99	0.996	0.572	0.987
I194	0.975	0.997	0.88	0.989
F195	0.53	0.997	0.753	0.995

Although binding site 8 is associated only with binding of CL-0—a nonphysiological charge state of CL—it provides some valuable insights into how the acyl chains of CL, regardless of charge state, interact with the surface of PR. BS8 is a 10 nm^2^ patch located at the interstitial region of the bilayer between helix F and G, with a large degree of overlap with BS3. Most notably, BS8 is comprised almost exclusively of nonpolar residues that significantly stabilize the CL-0 acyl chain (T188, I192, F195, G196, V229, L233, F234, and I237), with several per-residue residence times reaching up to 8 μs (Table S9). This strong interaction would seem to indicate that BS8 is only accessible to noncharged lipids like CL-0 or other nonpolar lipid tails.

## Discussion

Both proteorhodopsin and cardiolipin are ubiquitous in microbiota.^1−4^ PR is directly responsible for generating proton gradients across bacterial cell membranes, and CL is indirectly involved in proton pumping in prokaryotes—for example, glycocardiolipin interacts with the canonical microbial rhodopsin, bacteriorhodopsin, altering the behavior of protons along the membrane surface and in bulk periplasm after proton transport.^35^ Based on our simulation results, it appears that CL may also play a role in modulating the function of PR. Both BS3 and BS6 lie proximal to functional hot spots on PR—the Schiff base (BS3) and the EF loop (BS6). There is biological precedent for BS6 serving as a nexus for energy transfer to enhance efficiency of solar harvesting. Originally it was proposed that excitation-energy transfer (EET)—enhancement of photoactivation via the physical interaction of carotenoid light-harvesting antennae and retinal—mainly occurred in the xanthorhodopsin family^49^ and other halophilic bacteria.^50^ However, more recently, it was shown that the fenestration site that facilitates carotenoid binding is conserved across multiple families of rhodopsins, including PR.^51^ It appears that binding of CL-1 to PR at BS6 could potentially inhibit binding of carotenoids. Even though W149 in BS6 has statistically significant interactions with CL-1 (on the order of 100 ns) and is one of the conserved residues in the fenestration site, the additional residues in BS6 would actually prevent carotenoids from binding to PR, as shown by aligning the xandthorhodopsin structure to the structure of PR (Figure S5). What implications this has to the role of EET in PR function will need to be validated by experiment.

In addition to potential occlusion of carotenoid binding to the surface of PR, the binding of CL to BS6 may also influence color tuning of the protein. The A178R red-shifted mutant of PR lies in the EF loop and is flanked by several residues in BS6 (i.e., E170, G171, and N187). Although A178 lies distal from the retinal binding pocket (25 Å), the loop conformation has a direct effect on the arrangement of transmembrane helices that leads to control of light absorption.^9,52,53^ Stable binding of CL-1 could help stabilize PR in the ground state, effectively counteracting the destabilizing effects of substitutions at position 178. This characteristic would most likely be specific to the −1 charge state of CL, as both CL-0 and CL-2 would bind poorly to BS6, but for different reasons: the neutral headgroup of CL-0 would have to be exposed to bulk solvent and the highly charged headgroup of CL-2 has unfavorable electrostatic interactions with the largely nonpolar side chains of BS6 proximal to the EF loop.

BS3 is the most likely binding site for CL as it possesses distinct hydrophilic and hydrophobic patches that can take advantage of the dual topology of CL. The asymmetric charge distribution of the CL-1 headgroup as well as the phosphate moieties display lo ng–time scale interactions with the polar residues on the cytoplasmic end of helices F and G. Y186, N187, T188, W239, N240, and K244 form a shallow groove that allows for lateral movement of the CL-1 headgroup along the surface of PR. Although it was previously suggested that positively charged residues are a conserved motif for stabilization of the phosphate groups in CL^27^ and K244 in BS3 supports this hypothesis, the shorter per-residue residence time of K244 (29 ns for 10 mol %) suggests that the combined effect of the network of polar residues in the cytoplasmic half of BS3 are what lead to stable interactions between the headgroup of CL-1 and PR. In contrast, the hydrophobic residues in BS3 form noticeably longer per-residue interactions with the acyl tails of CL-1 regardless of mol % of CL, suggesting that this hydrophobic patch is conserved and an essential component to BS3. Visual inspection shows that this is largely due to shape complementarity, and the symmetric nature of the acyl chains in CL essentially doubles the probability that a long-lived interaction will occur with PR.

Although our study cannot directly validate the role of CL in the stabilization or destabilization of oligomers of PR, there are many examples in the literature showing how CL is directly involved in the aggregation of membrane proteins.^26,54^ Of the nine binding sites we identified, BS0, BS1, and BS2 are located proximal to helix A, which has been shown to be critical to tuning of oligomerization in PR.^12^ In addition to residue-specific controls of oligomerization of PR, membrane environment can modulate the degree of oligomerization,^10,55^ lending further support to the hypothesis that CL could influence function of PR. The salt bridge formed by E50, R51, and D52 between helix A of neighboring multimers acts as a switch between pentameric and hexameric states of PR.^12,14^ This salt bridge places helix A in close proximity to helices A, B, and C on the neighboring monomer. These multimeric interfaces could serve as additional binding sites for CL that would presumably stabilize a PR oligomer. The most likely candidates for a hybrid binding site are BS0 and BS2, as they have residues close to the oligomeric interface at the intracellular and extracellular sides of the membrane.

## Conclusions

Our computational study shows that the −1 charge state of cardiolipin is highly likely to be a functional ligand of green proteorhodopsin. Two potential binding sites of CL-1 were identified (BS3 and BS6), both with residence times several factors higher than the residence time of POPE and POPG. Both BS3 and BS6 display multiple features that support the hypothesis of CL-1 as a modulator of PR. First, they both have multiμs residence times, far greater than the residence times of other potential binding sites for biologically relevant lipids. Second, they are homologous to CL binding sites that have been previously identified on other membrane proteins. Finally, both binding sites are proximal to functional hotspots on PR. Specifically, BS3 has interactions with multiple residues that form the outer shell on the Schiff base end of the retinal binding pocket, and BS6 has direct contact with the EF loop which plays a role in the color tuning of PR photoabsorption. While our MD simulations provide some potentially promising indications of the role that CL plays in PR function, experimental studies will be necessary to validate our findings. Future computational studies will also focus on the role of CL in the oligomerization of PR, which is a critical aspect in the function of the vast majority of microbial rhodopsins.

## Supporting Information Available

Includes detailed information about cardiolipin binding times and binding sites, POPE and POPG lipid characteristics, and comparison between structures of PR and xanthorhodopsin (PDF)
