# Supplementary material for: Coarse-Grained Molecular Dynamics Simulations Reveal Potential Role of Cardiolipin in Lateral Organization of Proteorhodopsin
Source: Biochemistry. 2025 Mar 26;64(8):1887–94. doi: 10.1021/acs.biochem.4c00831 (PMC12004449; doi:10.1021/acs.biochem.4c00831)
Supplement: Supplementary file 1 — bi4c00831_si_001.pdf [file bi4c00831_si_001.pdf]

Supporting Information for "Coarse-grained molecular dynamics simulations reveal potential role of cardiolipin in lateral organization of proteorhodopsin"

Alexander Wroe[*], Eric Sefah[1,*], and Blake Mertz[2,*]

[*] C. Eugene Bennett Department of Chemistry, West Virginia University, Morgantown, WV, USA
[1] Current address: National Institutes of Health, Bethesda, MD, 20892 USA
[2] Current address: Alivexis, Inc., Cambridge, MA 02142 USA

Corresponding author: mertz@alivexis.com

This PDF file includes:

- Tables S1 to S9
- Figures S1 to S6

Table S1: Binding site statistics for CL-0 at 5 mol %.

site	restime/µs	K_{off}	R^2	duration/µs	occupancy/%	lipid count	SA[a]/nm^2
0	0.397	2.521	0.9988	0.021	86.166	1.588	18.800
1	0.114	8.774	0.9994	0.009	70.076	1.432	14.772
2	0.045	22.100	0.9924	0.004	3.068	1.001	5.359
3	0.069	14.582	0.9172	0.008	62.982	1.295	15.425
4	0.221	4.522	0.9987	0.012	49.244	1.236	15.659
5	0.322	3.106	0.9939	0.008	57.019	1.232	14.560
6	1.812	0.552	0.9714	0.033	95.709	2.022	18.907
7	0.002	659.827	0.9943	0.001	0.033	.667	1.075
8	4.603	0.217	0.9947	0.040	99.696	2.414	12.012

[a] Average surface area.

Table S2: Binding site statistics for CL-0 at 10 mol %.

site	restime/µs	K_{off}	R^2	duration/µs	occupancy/%	lipid count	SA[a]/nm^2
0	0.389	2.573	0.9996	0.020	96.546	2.410	17.778
1	0.115	8.676	0.9981	0.007	69.100	1.540	16.225
2	0.036	27.566	0.9978	0.003	10.678	1.021	7.352
3	0.067	14.860	0.9597	0.009	85.360	1.752	15.725
4	0.136	7.378	0.9987	0.013	84.889	1.619	15.396
5	0.179	5.587	0.9969	0.010	85.952	1.740	15.239
6	3.186	0.314	0.9724	0.028	100.000	3.372	2.520
7	0.003	310.198	1	0.002	1.512	1.014	5.475
8	8.000	0.059	0.9886	0.033	100.000	3.284	12.133

[a] Average surface area.

Table S3: Binding site statistics for CL-1 at 5 mol %.

site	restime/µs	K_{off}	R^2	duration/µs	occupancy/%	lipid count	SA[a]/nm^2
0	2.19	4.575	0.9993	0.016	75.770	1.314	17.880
1	0.197	5.066	0.9989	0.012	71.333	1.389	11.343
2	0.020	49.094	0.9572	0.004	6.199	1.007	9.731
3	3.647	0.274	0.8718	0.016	86.306	1.469	22.511
4	0.099	10.140	0.9992	0.011	44.782	1.132	14.293
5	0.182	5.495	0.9975	0.012	74.578	1.355	19.939
6	7.373	0.136	0.8822	0.048	99.075	2.209	16.646
7	0.004	225.504	0.9997	0.002	0.663	1.000	5.591

[a] Average surface area.

Table S4: Binding site statistics for CL-1 at 10 mol %.

site	restime/µs	K_{off}	R^2	duration/µs	occupancy/%	lipid count	SA[a]/nm^2
0	0.297	3.362	0.9979	0.013	93.519	2.031	17.995
1	0.332	3.008	0.9979	0.011	84.118	1.643	11.055
2	0.151	6.606	0.9977	0.008	67.752	1.502	18.674
3	1.263	0.792	0.9286	0.016	97.642	2.372	23.400
4	0.099	10.137	0.9704	0.013	70.112	1.402	13.417
5	0.191	5.235	0.9971	0.012	89.066	1.836	18.581
6	2.569	0.389	0.8957	0.032	99.741	2.952	18.474
7	0.008	128.458	0.9993	0.003	1.178	1.028	5.622

[a] Average surface area.

Table S5: Binding site statistics for CL-2 at 5 mol %.

site	restime/µs	K_{off}	R^2	duration/µs	occupancy/%	lipid count	SA[a]/nm^2
0	0.099	10.065	0.9987	0.008	37.279	1.121	13.660
1	0.206	4.854	0.9975	0.012	66.558	1.298	13.958
2	0.301	3.324	0.9980	0.013	67.904	1.274	18.638
3	0.325	3.080	0.9965	0.014	66.392	1.362	22.594
4	0.115	8.666	0.9981	0.009	37.479	1.111	17.419
5	0.099	10.055	0.9986	0.009	49.344	1.163	16.921
6	0.177	35.635	0.9955	0.011	40.457	1.176	16.780
7	0.010	98.296	0.9991	0.003	0.142	1.000	3.713

[a] Average surface area.

Table S6: Binding site statistics for CL-2 at 10 mol %.

site	restime/µs	K_{off}	R^2	duration/µs	occupancy/%	lipid count	SA[a]/nm^2
0	0.186	5.368	0.9972	0.008	77.732	1.573	15.989
1	0.306	3.267	0.9965	0.012	82.186	1.599	16.303
2	0.347	2.880	0.9968	0.013	91.297	2.026	25.062
3	0.584	1.712	0.9982	0.016	94.671	2.056	22.373
4	0.058	17.198	0.9978	0.007	49.061	1.227	13.496
5	0.141	7.111	0.9952	0.009	75.128	1.481	12.862
6	0.442	2.262	0.9927	0.010	65.592	1.380	15.700
7	0.008	122.518	0.9984	0.003	0.125	1.000	2.323

[a] Average surface area.

Table S7: Binding residence times of POPE to PR.

(µs)	BS0	BS1	BS2	BS3	BS4	BS5	BS6	BS7	BS8	BS9	BS10
max	0.047	0.064	0.107	0.101	0.095	0.049	0.158	0.225	0.087	0.083	0.034
avg.	0.034	0.052	0.058	0.066	0.049	0.034	0.049	0.077	0.030	0.048	0.034
min	0.021	0.041	0.039	0.035	0.026	0.003	0	0.025	0.001	0.002	0.034

Table S8: Binding residence times of POPG to PR.

(µs)	BS0	BS1	BS2	BS3	BS4	BS5	BS6	BS7	BS8	BS9
max	0.056	0.061	0.090	0.091	0.066	0.068	0.046	0.139	0.045	0.221
avg.	0.042	0.040	0.058	0.063	0.039	0.046	0.025	0.055	0.034	0.081
min	0.028	0.027	0.035	0.031	0.023	0.026	0.003	0.018	0.019	0.010

Table S9: Per-residue binding residence times of CL-0 to binding site 8.

BS8	10% CL-0		5% CL-0	
residue	time/µs	R^2	time/µs	R^2
T188	8.000	0.774	7.990	0.893
Y191	0.575	0.999	1.183	0.996
I192	8.000	0.886	8.000	0.875
F195	1.768	0.957	7.792	0.914
G196	8.000	0.883	8.000	0.874
V229	8.000	0.874	8.000	0.875
N230	0.855	0.998	1.211	0.999
L233	6.455	0.972	8.000	0.882
F234	8.000	0.886	8.000	0.878
I237	8.000	0.763	3.267	0.966

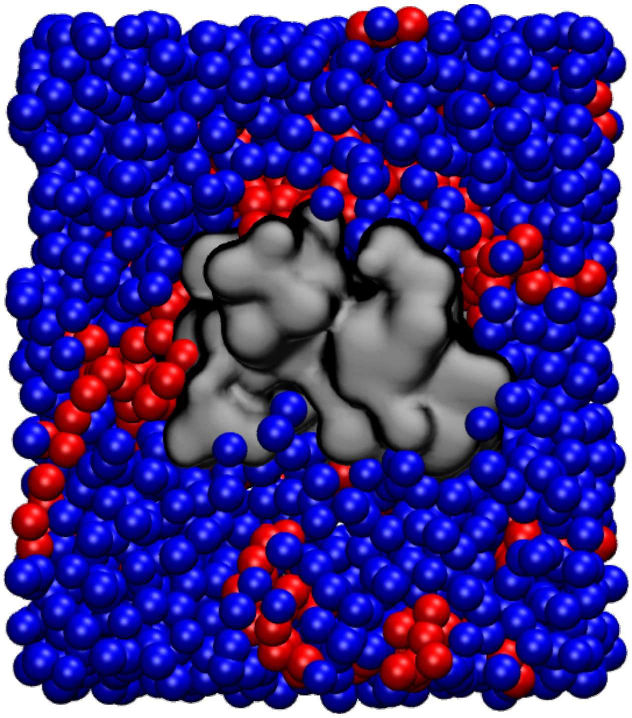

Figure S1. Representative snapshot of equilibrated proteolipid system. *Gray*: proteorhodopsin; *blue*: POPG and POPE; *red*: cardiolipin.

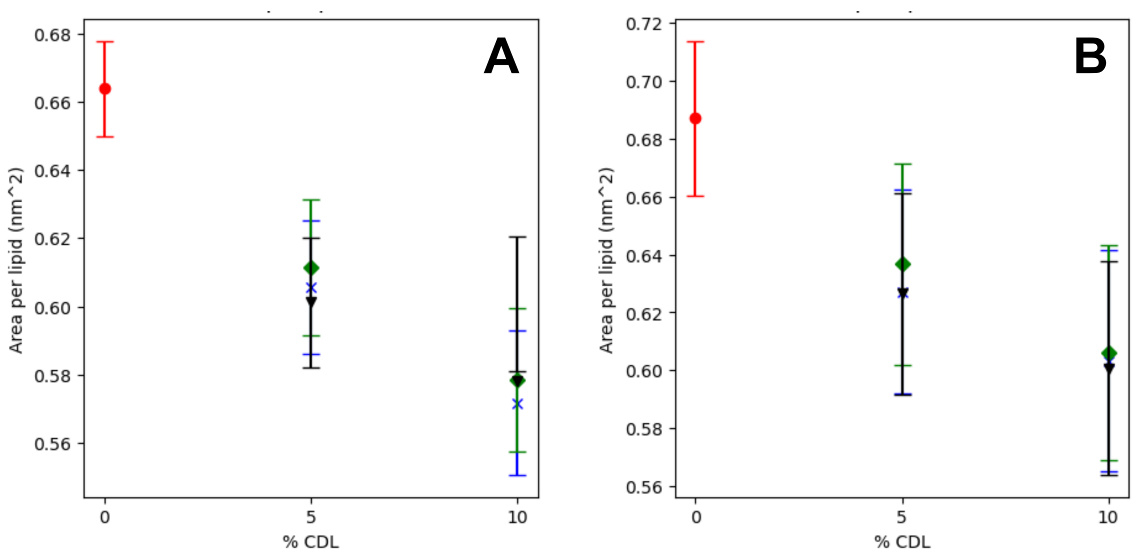

Figure S2. Cardiolipin increases lateral packing of the lipid bilayer. A) Average Area per lipid and standard deviation of POPE as a function of mol % of CL. **B)** Average Area per lipid and standard deviation of POPG as a function of mol % of CL. Red circle: no CL; blue cross: CL-0; green diamond: CL-1; black triangle: CL-2.

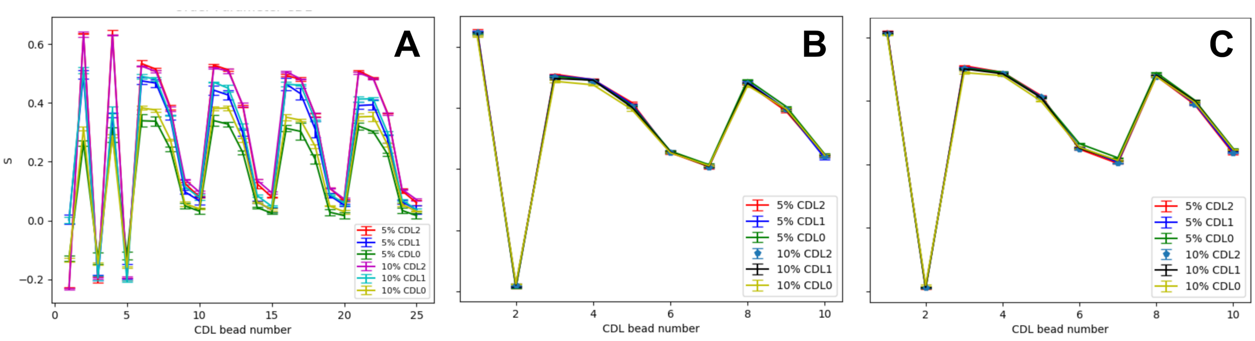

Figure S3. Charge state of CL has differential ordering effects on the bilayer. A) Pseudo order parameters for CL as a function of mol % of CL (5% and 10%) and charge state (CL-0, CL-1, and CL-2). B) Pseudo order parameters for POPG as a function of mol % of CL (5% and 10%) and charge state (CL-0, CL-1, and CL-2). C) Pseudo order parameters for POPE as a function of mol % of CL (5% and 10%) and charge state (CL-0, CL-1, and CL-2).

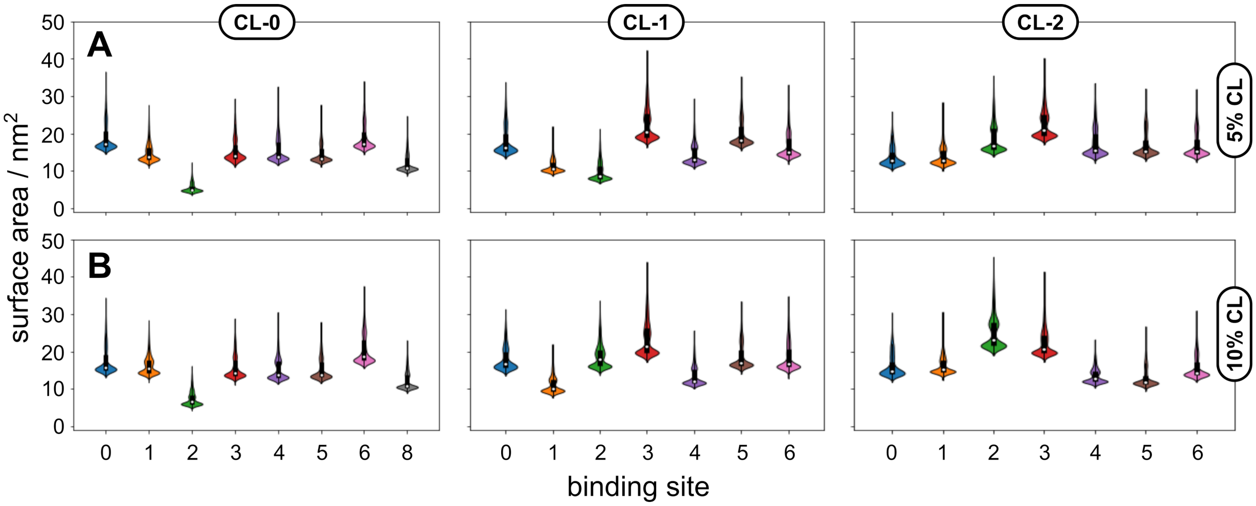

Figure S4. Charge asymmetry of cardiolipin generates variability in the surface area of binding sites on PR. A) Surface area for binding sites on PR with respect to the -0 (left), -1 (middle), and -2 (right) charge states with a 5% mole fraction of CL. **B)** Surface area for binding sites on PR with respect to the -0 (left), -1 (middle), and -2 (right) charge states with a 10% mole fraction of CL. (Binding site 7 was omitted for CL-0 because it was significantly smaller than the rest of the binding sites.)

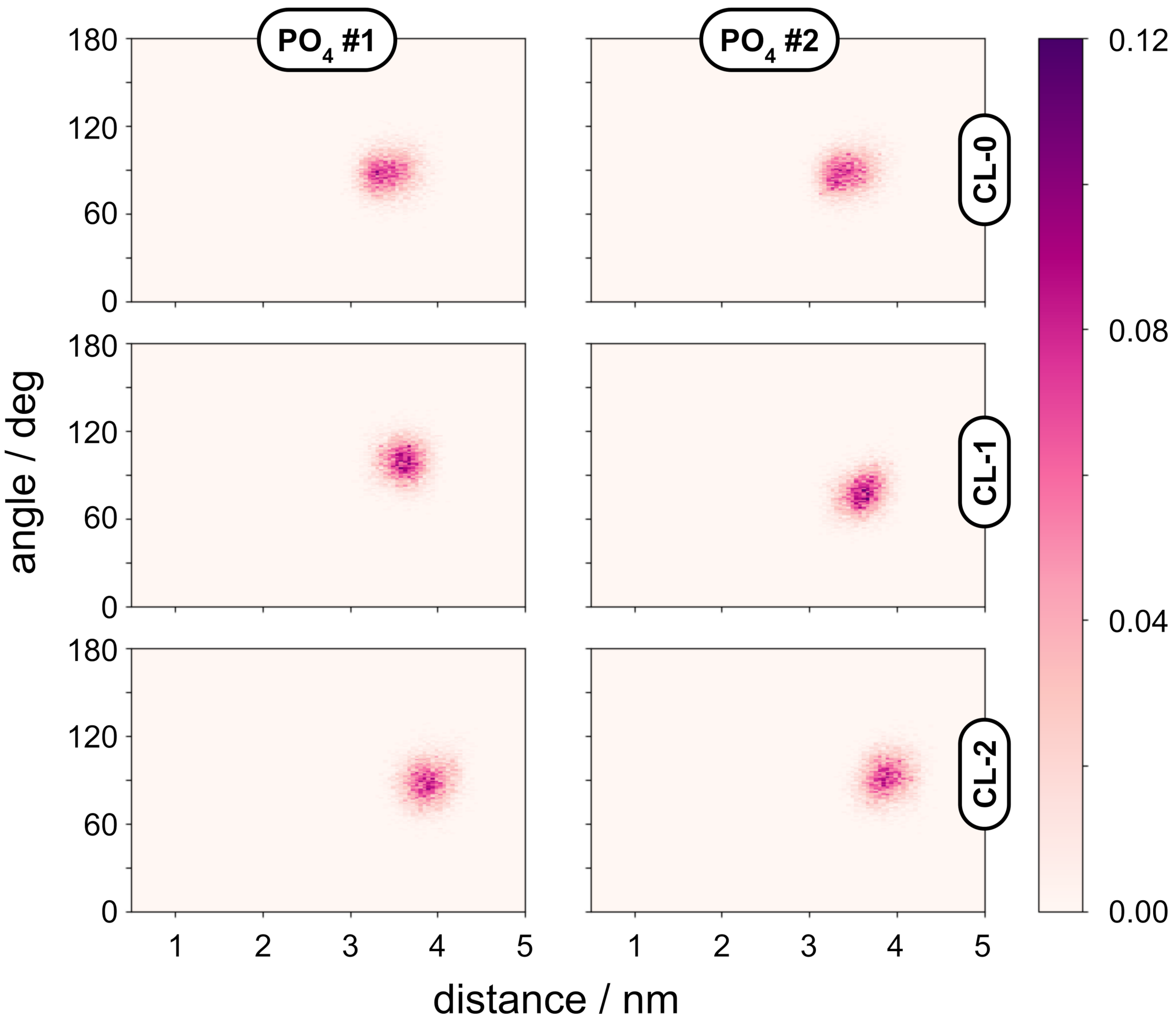

Figure S5. Asymmetric headgroups lead to a shift in the orientation of CL to preferred PR binding sites. Distribution of average distance between the two phosphate beads on the CL headgroup and the Schiff base of PR (K231) versus the angle formed between the reference residue, the corresponding phosphate bead, and K231 at 10 mol% CL. For CL lipids in the upper leaflet, the reference residue was A114 in PR, and for CL lipids in the lower leaflet, the reference residue was N220 in PR.

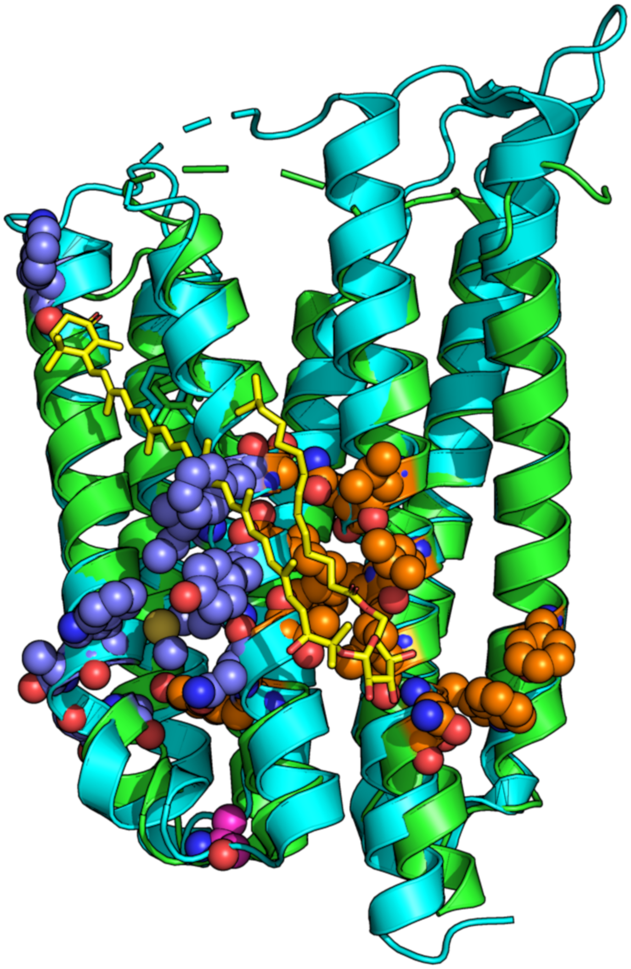

Figure S6. CL binding could interfere with putative excitation-energy transfer in PR. Overlay of xanthorhodopsin (XR) structure (PDB 3DDL) with green PR (PDB 7B03). The salinixanthin antenna of XR interacts with the beta-ionone ring of retinal. Both BS3 and BS6 have areas of overlap with the region of the salinixanthin-retinal interaction; binding of CL (in particular CL-1) would prevent energy transfer from taking place. *Green:* PR; *cyan:* XR; *sticks:* salinixanthin and retinal; *orange spheres:* BS3; *purple spheres:* BS6.
